# The Applications of Nanopore Sequencing Technology in Animal and Human Virus Research

**DOI:** 10.3390/v16050798

**Published:** 2024-05-16

**Authors:** Chun-Miao Ji, Xiao-Yin Feng, Yao-Wei Huang, Rui-Ai Chen

**Affiliations:** 1 Zhaoqing Branch Center of Guangdong Laboratory for Lingnan Modern Agricultural Science and Technology, Zhaoqing 526238, China; jichunmiaomiao@163.com (C.-M.J.); xiaoyin_f@163.com (X.-Y.F.); 2 College of Veterinary Medicine, South China Agricultural University, Guangzhou 510642, China; yhuang@zju.edu.cn; 3 Department of Veterinary Medicine, Zhejiang University, Hangzhou 310058, China

**Keywords:** nanopore sequencing, viruses, applications, chemical modifications, genome assembly

## Abstract

In recent years, an increasing number of viruses have triggered outbreaks that pose a severe threat to both human and animal life, as well as caused substantial economic losses. It is crucial to understand the genomic structure and epidemiology of these viruses to guide effective clinical prevention and treatment strategies. Nanopore sequencing, a third-generation sequencing technology, has been widely used in genomic research since 2014. This technology offers several advantages over traditional methods and next-generation sequencing (NGS), such as the ability to generate ultra-long reads, high efficiency, real-time monitoring and analysis, portability, and the ability to directly sequence RNA or DNA molecules. As a result, it exhibits excellent applicability and flexibility in virus research, including viral detection and surveillance, genome assembly, the discovery of new variants and novel viruses, and the identification of chemical modifications. In this paper, we provide a comprehensive review of the development, principles, advantages, and applications of nanopore sequencing technology in animal and human virus research, aiming to offer fresh perspectives for future studies in this field.

## 1. Introduction

The viruses known to date exhibit remarkable diversity and complexity, and it is estimated that many more viruses remain to be discovered globally. Animal viruses, such as African swine fever virus (ASFV) [1], porcine reproductive and respiratory syndrome virus (PRRSV) [2], porcine epidemic diarrhea virus (PEDV) [3,4], Newcastle disease virus (NDV) [5], infectious bronchitis virus (IBV) [6], and others, cause the death of animals and lower the quality of animal products, which results in great economic losses in the livestock industry. ASFV, in particular, is highly pathogenic and spreads quickly and efficiently between pig herds, leading to pandemic outbreaks with mortality rates approaching 100% [7,8]. Certain viruses, such as influenza A virus (IAV) and the severe acute respiratory syndrome coronavirus 2 (SARS-CoV-2), can transmit from animals to humans and cause zoonosis, endangering human health and life. IAV, capable of genetic reassortment and the emergence of highly pathogenic variants, is responsible for seasonal and global influenza pandemics that result in high morbidity and mortality rates in both humans and poultry [9,10]. The coronavirus disease 2019 (COVID-19), caused by SARS-CoV-2, has rapidly spread worldwide through contact and respiratory droplets, resulting in more than 770 million confirmed cases and 6.9 million deaths up to September 2023 (https://data.who.int/dashboards/covid19/cases?n=c (accessed on 12 September 2023)) [11,12]. SARS-CoV-2 is believed to have a zoonotic origin, although the specific animal reservoir has not been identified. Reports suggest that SARS-CoV-2 can also infect other animals, such as domestic cats, dogs, etc. [13,14,15,16]. Given the high risk that viruses pose to both human and animal health, the development of a rapid and convenient detection method is crucial in monitoring, controlling, and researching viruses.

Nanopore sequencing technology has been extensively employed in researching since 2014, when MinION sequencer, developed by Oxford Nanopore Technologies (ONT), was made available to early-access users [17]. To date, nanopore sequencing has found applications in the identification of clinically relevant viruses and structural variations [18,19,20,21], the assembly of new reference genomes [22,23], the detection of DNA and RNA modifications [24,25], outbreak surveillance [26,27], on-site metagenomics research [28], and more [29,30,31]. Distinct from next-generation sequencing (NGS), also known as short-read sequencing, nanopore sequencing technology is capable of sequencing single long strands of DNA or RNA, thereby enhancing the quality and throughput of sequencing [32]. It offers a number of benefits over traditional methods, including reduced turnaround times and lower equipment costs [33]. The technology also provides unique advantages in terms of convenience, real-time genomic surveillance, and analysis [34,35], which are not constrained by geographical location or time. Thanks to its versatility and adaptability, nanopore sequencing has been widely applied in the field of virus research.

## 2. Nanopore Sequencing Technology

### 2.1. Development

**Figure 1:**
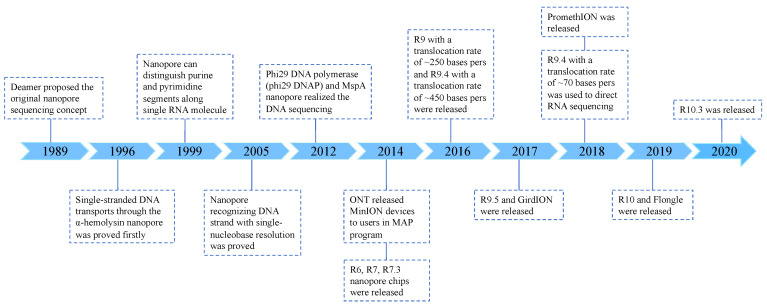
The development of nanopore sequencing. Since the concept of nanopore sequencing was proposed in 1989, the technology has undergone significant development over a period of 30 years. The commercialization of nanopore sequencing kits began in 2014, and they have since been widely used in multiple fields.

The original concept of nanopore sequencing was introduced by Deamer in 1989 (Figure 1) [36]. In 1996, Deamer, along with Daniel Branton and colleagues, was the first to demonstrate the transport of single-stranded DNA through the α-hemolysin nanopore [37]. It was further demonstrated in 1999 that nanopores could distinguish between purine and pyrimidine segments along a single RNA molecule [38]. Shortly thereafter, Bayley and Ghadiri demonstrated that nanopores could recognize DNA strands with single-nucleotide resolution [39,40,41,42]. The results from the phi29 motor combined with the *Mycobacterium smegmatis* porin A (MspA) nanopore contributed to the advancement of nanopore sequencing technology [43]. In 2014, ONT released the MinION devices to early-access users as part of the Mapper Access Program (MAP), signifying the commercialization of nanopore sequencing [36]. ONT has since continually improved and upgraded the technology, releasing eight versions of the nanopore chip and introducing new devices such as GridION and PromethION [32,44].

### 2.2. Principle

**Figure 2:**
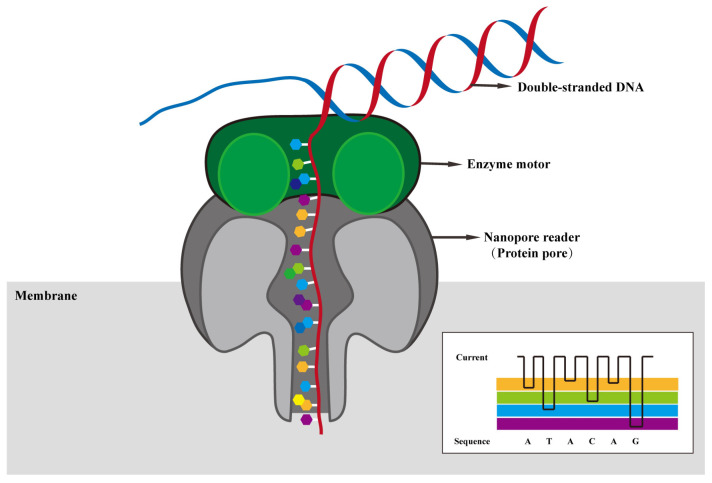
The principles of nanopore sequencing. During nanopore sequencing, as DNA or RNA molecules pass through the nanopore, an enzyme motor controls the translocation of the single strand, resulting in distinctive signals of ionic current fluctuations that are detected by sensors within the nanopore. These current signal datasets are then processed and translated into nucleic acid sequences by bioinformatics software.

Nanopore sequencing technology leverages the distinct electrical signals produced by different nucleotide bases to identify their types. Nanopores, with their narrow diameters, permit the passage of only a single purine or pyrimidine nucleotide at a time [37]. During the sequencing process, double-stranded DNA is unwound by the action of a motor protein. The resulting single-stranded DNA and RNA molecule are then propelled through the nanopores by an applied electric field [45]. When negatively charged biomolecules pass through nanopores, the ionic current fluctuates and different bases produce distinct changes in current [46]. These changes of current signal can be captured and analyzed using computer-aided tools [46,47]. Nanopore sequencing allows direct DNA or RNA sequencing without the need for DNA synthesis reactions because nucleotides are recorded directly from the current signal. The current signal is converted into a base sequence through basecalling (Figure 2).

Nanopores can be categorized into two types: solid-state nanopores and biological nanopores. Biological nanopores can be either artificially created through genetic engineering or derived from natural protein molecules [48]. They are commonly employed for gene sequencing [49], disease diagnosis [50], and protein sequencing [51] of viruses.

### 2.3. Methods

Viral nucleic acids, including genomic DNA or RNA, as well as amplified DNA and transcripts, can be detected by a nanopore sequenator. The nanopore sequencing processes encompasses library preparation, sequencing, and bioinformatics analysis. Currently, ONT has introduced numerous library preparation strategies and a variety of kits suitable for virus research.

#### 2.3.1. DNA Sequencing

DNA is extracted from samples using DNA extraction kits, such as the QIAamp DNA Micro Kit (QIAGEN, Hilden, Germany). Subsequently, the length, quantity, and purity of the DNA are assessed, which is crucial for guaranteeing the success of the experiment. The choice of library preparation kit is determined based on the specific objectives of experiment and the actual conditions (Table 1).

**Figure 3:**
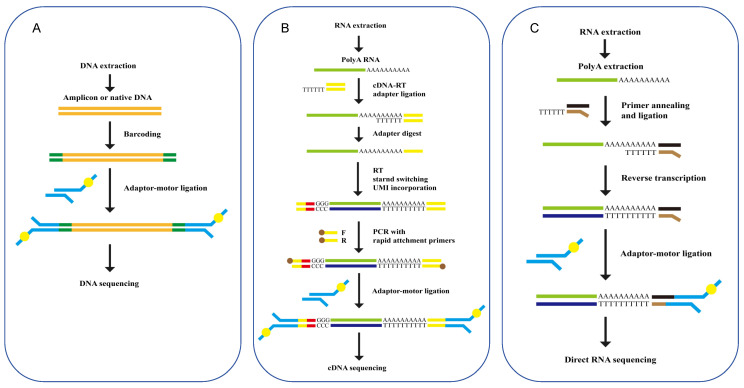
The principles of nanopore library preparation for viruses. (**A**) DNA library preparation. DNA library preparation kits, such as SQK-RPB004, SQK-LSK114 and SQK-LSK109 (Oxford Nanopore Technologies, Oxford, England), facilitate the preparation of DNA libraries from either amplicon or native DNA inputs. These molecules are barcoded using a barcoding kit through PCR or rolling circle amplification (RCA). (**B**) cDNA library preparation. The cDNA-PCR sequencing kit SQK-PCB114 (Oxford Nanopore Technologies, Oxford, England) uses a strand-switching method with poly (A) RNA as a template to generate libraries and sequences. During the strand-switching, a unique molecular identifier (UMI) is incorporated into the double-strand cDNA. Subsequently, the double-stranded cDNA is amplified by PCR using primers with 5′ tags. Finally, sequencing adapters are ligated to the amplified cDNA. (**C**) Direct RNA library preparation. Direct RNA library preparation kits, such as SQK-RNA004 (Oxford Nanopore Technologies, Oxford, England), enable the direct submission of either poly (A)-tailed RNA or total RNA. A reverse transcription step is recommended to enhance the sequencing output. Following this, an adaptor is ligated to the RNA for subsequent nanopore sequencing. Only native RNA is able to pass through the nanopore sensor.

Beyond targeted sequencing, the workflows for other sequencing kits predominantly involve fragmentation, end preparation, and adapter ligation (Figure 3A). The workflows for the rapid PCR barcoding kit and the 16S barcoding kit also include an amplification step. Additionally, specialized Cas9 ribonucleoprotein (RNP) complexes are required for the preparation of the Cas9 sequencing kit.

#### 2.3.2. RNA Sequencing

RNA extraction and quality control procedures are analogous to those used for DNA, albeit with modifications to account for the shorter half-life and structural differences of RNA. RNA sequencing can be conducted through either cDNA-PCR sequencing or direct RNA sequencing, with the choice of method dependent on the specific experimental aims and the context of the study (Table 2). Unlike cDNA-PCR sequencing, which involves the conversion of RNA to cDNA followed by PCR amplification (Figure 3B), direct RNA sequencing can be applied to the RNA itself (Figure 3C). This approach ensures that the read length reflects the actual length of the RNA molecules in the sample.

RNA viruses, which typically lack poly(A) tails, are not amenable to sequencing using standard methods. However, a number of strategies have been developed to facilitate the addition of poly(A) tails to total RNA prior to library preparation. One such strategy involves the use of poly(A) polymerase derived from *E. coli*.

#### 2.3.3. Bioinformatics Analysis

The POD5 or fast5 files are acquired through sequencing, and these files serve as the raw data for subsequent analysis. The workflow for analyzing viruses primarily involves four key processes: (1) converting the current signals into a base sequence through a process known as basecalling, (2) performing quality control on the sequence data to ensure accuracy and reliability, (3) aligning the sequences with the reference sequences to identify variations, and (4) conducting additional analyses, such as determining methylation patterns. A number of software tools have been developed specifically for the nanopore sequencing data of viruses (Table 3).

### 2.4. Advantages

Since the release of MinKNOW by ONT in 2004, there has been a significant surge in popularity of nanopore sequencing technology and its applications in both basic and applied research, thanks to its numerous unique advantages.

Nanopore sequencing stands out for its real-time capabilities and high efficiency. It is able to detect pathogens and generate sequencing data in a much shorter timeframe compared to NGS, positioning it as a more effective option for rapid genetic profiling [52,53]. The library preparation process for nanopore sequencing is remarkably simple, requiring only 10 min when using a rapid barcoding kit [33,54]. This efficiency is further enhanced by direct RNA sequencing, which eliminates the need for reverse transcription, PCR amplification, and other steps, thereby saving both time and costs. Additionally, the MinION flow cell enables the parallel sequencing of multiple samples and the generation of data in real time. The sequencers can be easily connected to a computer via a port, allowing researchers to monitor and analyze raw data in real time as sequencing occurs [55,56,57]. The portability of nanopore sequencing even allows for its use in microgravity conditions [58].

Nanopore sequencing also offers greatly extended read lengths, surpassing those achievable with second-generation Illumina sequencing. In 2018, Alexander Payne demonstrated read lengths of up to 2.273 Mb [59]. This capability to generate long reads has enhanced the accuracy of identifying complex repetitive and/or rearranged structures in DNA/RNA molecules, including plasmids, and has facilitated the detection of a full spectrum of structural variations [60,61]. It has also enabled the complete assembly of genomes for complex mammals and microorganisms [62,63]. Both the nanopore and PacBio sequencers are capable of producing long read sequences. However, the nanopore platform has the potential to generate ultra-long reads beyond 100 Kb, which could revolutionize research in genomics and proteomics. Furthermore, adaptive sampling, a pioneering feature of nanopore sequencing, adjusts sequencing parameters to target specific genomic regions, thereby improving coverage and the efficient use of sequencing resources [64].

The convenience and cost-effectiveness of nanopore sequencing are also significant benefits. The MinION device is a lightweight and portable sequencer that offers unparalleled convenience for transportation and operation in remote and field environments, a feature not provided by PacBio or Illumine sequencers. ONT has made efforts to reduce costs, with the MinION device priced at just USD1000 and the Flongle, which allows for one-time sequencing on small flow cells, at only USD100 [52]. Several studies have shown that the ONT platform can be more cost-effective than the PacBio or Illumina platforms [65,66]. Nanopore sequencing has been utilized for species identification in the field [67,68], enabling rapid in situ biodiversity assessments [69,70], monitoring of endangered species, and surveillance of invasive species [71].

One of the most exciting attributes of nanopore sequencing is its ability to sequence direct RNA. In contrast, although PacBio sequencing can handle RNA inputs, it does not offer direct RNA sequencing. Different from traditional methods, direct RNA sequencing bypasses the steps of reverse transcription and PCR amplification, which can introduce errors [72]. This direct approach accurately captures RNA modifications, providing insights into viral epigenetics [73,74]. When combined with the ability to generate long reads, direct RNA sequencing allows researchers to obtain full-length transcripts and gain a comprehensive view of the subgenome landscape of viruses [75].

## 3. Applications

### 3.1. Diagnosis and Monitoring of Viruses

In recent years, emerging and re-emerging viruses have posed a significant challenge to global public health due to increased globalization of economies and agricultural trade. Nanopore sequencing, with its unique capabilities for real-time analysis and portability, has emerged as critical in the timely diagnosis of virus strains and monitoring of the spread of viral infections. This technology significantly aids in the understanding of disease epidemiology, facilitating effective prevention strategies and informing treatment decisions. Increasingly, research is being published that employs nanopore sequencing to detect and analyze viruses that cause diseases in both humans and animals. This includes the study of DNA viruses, such as African swine fever virus (ASFV) and monkeypox virus (MPXV), as well as RNA viruses including influenza A virus (IAV), human metapneumovirus (HMPV), and Ebola virus (EBOV).

ASFV is a prime example of a highly lethal animal virus, with a mortality rate reaching 100% and no vaccines or treatments currently available [76]. Understanding the epidemic trends of ASFV is crucial to preventing its spread. Researchers initially employed nanopore sequencing to perform genome-wide analysis on an ASFV clinical sample collected in Zhuhai, China, uncovering that the genome sequence of this strain belonged to genotype II ASFV, identical to the classic ASFV isolate Georgia 2007/1 and the currently circulating strains SY18, AnhuiXCGQ, and HLJ18 in China [77]. Subsequently, in 2019, Jan et al. produced a high-quality whole-genome sequence of ASFV Georgia 2007/1 using the Rapid Barcoding Sequencing Kit (ONT, Oxford, UK) and Illumina sequencing, and they corrected 71 sequencing errors in the ASFV Georgia 2007/1 whole-genome sequence using Glimmer 3 [78]. These studies have provided more accurate information about the AFSV genome for future research. Simultaneously, the ability to generate data in real time is essential to the prompt analysis of high-consequence pathogens during an investigation. By leveraging the MinION rapid sequencing kit and the self-developed ASF-FAST software, Vivian K et al. were able to rapidly characterize and differentiate four distinct ASFV genome sequences from blood samples of infected pigs in real time [79]. These findings suggest that nanopore sequencing has the potential to uncover a wider array of ASFV genotypes, thereby laying the foundation for tracing the virus’s dissemination.

Monkeypox, a zoonotic disease caused by MPXV, a double-stranded DNA virus, has emerged as a significant global health concern [80,81]. There is an increasing mutation rate in human monkeypox [82,83], leading to outbreaks in more densely populated areas. Whole genome sequencing is essential for researching and monitoring MPXV. Balázs Kakuk and companions have sequenced the transcriptome of MPXV and the infected cells by cDNA sequencing and direct RNA sequencing with nanopore technology [84]. This research provides an LRS (long read sequencing) dataset, comprising nearly 1.5 million viral sequences, which facilitates comprehensive and temporal analysis of the monkeypox transcriptome and the impact of viral infection on host gene expression. In 2023, the potential transmission routes and mutational patterns of human monkeypox virus 1 (hMPXV1) were identified through rapid targeted nanopore sequencing [85]. Numerous studies have shown that nanopore technology enables researchers to gain a thorough understanding of the MPXV genome, laying the groundwork for real-time global monitoring of monkeypox virus lineages and outbreaks.

The influenza virus poses a great challenge to global public health due to its high degree of variability and pathogenicity, its substantial zoonotic reservoir, and its potential to cause pandemics [86]. Several studies have gradually uncovered the genetic complexities of IAV through nanopore sequencing, which has enhanced the ability to monitor and prevent the spread of IAV. Notably, Kuiama and colleagues were the first to employ metagenomic nanopore sequencing on clinical respiratory samples from the UK, successfully detecting the influenza virus and reconstructing complete virus genomes [49]. Subsequent studies identified 40 H1 and 19 H3 of hemagglutinin (HA) subtypes, and detected an S331R mutation in the H3N2 reassortant of IAV from 180 respiratory samples from a UK hospital during the 2018/19 influenza season using ONT metagenomic sequencing [87]. Rambo-Martin et al. utilized nanopore sequencing to identify a cluster of IAV in swine that exhibited genetic differences from candidate vaccine viruses, and developed a portable IAV sequencing and analysis platform named Mia (mobile influenza analysis) [88]. This platform was instrumental in identifying the predominant A virus (H1N2) found in exhibition pigs that had been transmitted to humans who had contact with the pigs at fairs in July and August 2018 (https://gis.cdc.gov/grasp/fluview/Novel_Influenza.html (accessed on 1 January 2024)). Clinically, rapid nanopore sequencing has become an invaluable tool for genotypic resistance detection and real-time monitoring of IAV within hospitals, greatly aiding in the efficient management of outbreaks [89]. Moreover, research has demonstrated that the NS and PB2 genes have the potential to serve as new targets for detecting both human and avian IAV via nanopore sequencing [90].

HMPV, first identified in the Netherlands in 2001, is a pathogen that can seriously harm the human respiratory system, exhibiting a high incidence during the spring and summer months. The advent of nanopore metagenomic sequencing has facilitated the diagnosis of HMPV infections. Utilizing this technology, a study was conducted with 25 clinical samples from 2017 to 2019 to investigate HMPV, firstly reporting the full-length genomes of HMPV from the UK and identifying genomes that belong to a unique genetic group within the A2b sublineage associated with nosocomial transmission among hematology patients [91]. Furthermore, nanopore sequencing offers novel insights into the evolutionary dynamics, genetics, and epidemiology of viruses. Through phylogenetic analysis complemented by nanopore sequencing, the study tracked the genetic lineages of HMPV over a period from 2005 to 2021, uncovering the mutation patterns and evolution of HMPV in Netherlands [92].

The use of nanopore sequencing has also extended to the analysis of EBOV genomes, one of the deadliest viruses known to humankind. A total of 142 EBOV samples collected from Guinean patients between March and October 2015 were sequenced, monitored and real-time analyzed using the MinION platform [34]. The analysis revealed two predominant lineages, named GN1 and SL3, which were also identified in Sierra Leone in early 2015, suggesting a pattern of transmission between the two countries. This study underscores the feasibility of implementing real-time genomic surveillance in resource-constrained environments, highlighting the potential for rapid deployment of nanopore sequencing to monitor and contain outbreaks.

### 3.2. Identification of Unknown Viruses

Nanopore sequencing, with its distinctive ability to perform long-read sequencing, enables discovering the lengthy and previous unknown genomic fragments within complex nucleic acid samples. This capability proved crucial in the characterization of emerging pathogens, such as the SARS-CoV-2 coronavirus, which was first identified in late 2019 and went on to cause the COVID-19 pandemic, posing a significant threat to human health and safety. Nanopore sequencing has emerged as a vital tool for quickly determining the genomic traits and origins of SARS-CoV-2.

Between January and March 2020, 29 complete genome sequences of SARS-CoV-2 were successfully obtained from clinical specimens collected in Hangzhou, through an efficient 8 h workflow. In addition, 33 variations were identified and analyzed within five coding sequences (CDSs) and the 5′ untranslated regions [93]. Phylogenetic analysis indicated that the majority of these cases originated from Wuhan, China, with some instances being traceable to imported cases from various other countries.

Real-time whole-genome sequencing (WGS) has been used to distinguish chronic infections from reinfections of SARS-CoV-2 and evaluate the reduced susceptibility of treatments due to the emerging mutations conferring resistance [94]. These results provide guidance for the subsequent therapeutic strategies of SARS-CoV-2. Nanopore WGS data have been pivotal in identifying in-hospital transmission and potential outbreaks of SARS-CoV-2, demonstrating that the source of infection is not always community-based but could also originate within hospitals [95]. These findings underscore the potential of nanopore technology over traditional NGS methods for investigating SARS-CoV-2, enabling prompt adjustments to infection prevention strategies and treatment modalities.

### 3.3. Transcriptome Assembly and Detection of Novel Transcript and Splice Variants

Nanopore sequencing is capable of reading full-length transcripts, making it an invaluable tool for genome assembly of viruses and the discovery of new variants. Due to its ability to provide comprehensive and accurate genomic information about various viruses, nanopore sequencing greatly facilitates the identification of new mutations and subtypes. This, in turn, offers fresh insights into the pathogenic mechanisms of viruses and paves the way for more effective vaccine development.

The hepatitis D virus (HDV) is a pathogen that can coinfect with hepatitis B virus (HBV), potentially leading to severe liver disease and even live cancer. The genetic sequencing of HDV is challenging due to its high genetic variability and complex genome structure. However, by combining nanopore long-read sequencing with a sequencing analysis pipeline called VIRiONT, the full-length HDV genomes were successfully amplified and sequenced in a single fragment, enabling the rapid identification of HDV genotypes and subtypes [96]. This method led to the discovery of a new subtype of HDV genotype 1, termed s22, which primarily comprises HDV sequences from Cameroon. It is evident that nanopore sequencing can accurately determine the genotypes and subgenotypes of HDV, aiding in a deeper understanding of HDV pathogenesis and treatment strategies.

The Varicella zoster virus (VZV) is a human-specific DNA virus that causes chickenpox and zoster, and understanding its pathogenic mechanism has been a challenging task. While previous studies have typically focused on investigating the pathogenic mechanism of VZV at the protein level [97], nanopore technology has enabled research into the virus’s RNA landscape. In 2018, István Prazsák and colleagues conducted a comprehensive analysis of the lytic transcriptome of VZV using nanopore MinION cDNA sequencing, identifying 114 novel transcripts, including mRNAs, non-coding RNAs, polycistronic RNAs, and complex transcripts [98]. Additionally, the researchers discovered 10 novel spliced transcripts and 25 novel transcription start site isoforms and transcription end site isoforms, as well as a novel class of transcripts named ncroRNAs.

Pseudorabies virus (PRV), a member of the *Alphaherpesvirinae* subfamily, along with VZV, causes severe nervous system disorders and a high mortality rates in young piglets, while leading to respiratory issues, growth stunting, and reproductive failures in adult pigs. These impacts result in substantial economic losses for pork producers globally [99]. PRV possesses a substantial double-stranded DNA genome and a correspondingly intricate transcriptome. Utilizing the nanopore, PacBio, and Illumina platforms, research has revealed 19 novel putative protein-coding genes, as well as three previously unidentified complex transcripts (UL32-35-C, UL20-21-C, and ul18-15d-17-16-C) within PRV. Additionally, 121 novel transcriptional overlaps were identified on the PRV genome [100]. These findings significantly enhance our comprehension of the transcriptional complexity and landscape of herpesviruses.

Adenovirus is a significant human pathogen that can cause a wide range of inflammatory responses of various body organs, including myocarditis, gastroenteritis, meningoencephalitis, and the inflammation of the respiratory tract [101,102]. It has been known to trigger local outbreaks and, in some cases, result in mortality, posing a threat to human health [103,104]. Researchers have employed nanopore sequencing technology to elucidate the genomic details of adenovirus. In 2021, the intricate transcriptome of human adenovirus type 2 (Ad2) was revealed through nanopore direct RNA sequencing, which led to the discovery of approximately 900 alternatively spliced mRNAs. This included 800 new alternative spliced mRNAs and 14 novel transcripts [105]. Additionally, in 2022, long-read direct RNA sequencing identified 35 new viral transcript isoforms of adenovirus serotypes 5 (Ad5), including 14 new splice junctions, 6 novel ORF-containing transcripts, and 15 transcripts that alter protein function by truncating or fusing with the canonical ORFs [106]. Notably, the novel transcript E4orf6/DBP, which is formed by the fusion of the N-terminus of E4orf6 to the downstream DBP ORF, has been shown to positively impact cell-to-cell spread and cell lysis.

The SARS-CoV-2 virus, with its intricate architecture of multiple nested subgenomic RNAs (sgRNAs), presents a complex transcriptomic profile that has proven challenging to fully elucidate. The short sgRNAs have, until recently, been difficult to sequence accurately, which has hindered reliable identification and quantification of the full-length coding transcripts. Based on nanopore sequencing, nanopore recappable sequencing (NRCeq) was used to identify the full-length RNA transcripts of SARS-CoV-2 and assemble the complete annotation of the sgRNAs [107]. As a result, 14 canonical sgRNA transcript models with lengths ranging from 370 to 8374nt were assembled by minimap2 and Pinfish. Additionally, seven non-canonical sgRNAs that resulted from independent transcription events or the recapping of 3’fragments of longer sgRNAs were assembled. The dynamic landscapes of SARS-CoV-2 subgenomes and their regulatory features were analyzed using NGS short-read and nanopore long-read poly(A) RNA sequencing. This research has revealed that the complex transcriptome structure of SARS-CoV-2 contains various types of full-length subgenomes [75]. Moreover, bidirectional template switches resulting from RNA-RNA interaction patterns have been observed. These switches play a significant role in generating the diversity of SARS-CoV-2 subgenomes, contributing to the virus’s complexity.

Nanopore sequencing has been used to detect single nucleotide variants (SNVs) in SARS-CoV-2. Studies have indicated that ONT sequencing is capable of accurately identifying within-specimen SNVs with frequencies ranging from 40% to 80%, yet it typically fails to detect the indels or rare SNVs (<40%) [108]. Additionally, structure variants have also been evaluated in SARS-CoV-2 and 16 candidate deletions ranging from 15 to 1840 bp were identified across various genes, including *S*, *M*, *N*, *ORF3*, *ORF6*, *ORF8*, and *orf1ab*. This research highlights the reliability of ONT sequencing in detecting large deletions and the common and varied occurrence of structural variations within the SARS-CoV-2 genome.

### 3.4. Analysis of Chemical Modifications

The chemical modification of nucleotides plays an essential role in the biological processes of various viruses and their eukaryotic host cells. Multiple modifications, including pseudouridine, N5-methylcytidine (m^5^C), 2′-O-methylation (Nm), N6-methyladinosine (m^6^A), N1-methylguanosine (m^1^G), and N4-acetylcytidine (ac^4^C), among others, have been identified within viral genomes and have been shown to perform different important functions in the viral life cycle [109,110]. Nanopore sequencing allows the direct sequencing of DNA and RNA molecules without the need for reverse transcription and amplification, enabling the detection and analysis of DNA and RNA modifications directly. With the advancement of nanopore sequencing, more and more analysis tools and software have been published and applied [111,112]. To date, numerous studies have utilized nanopore direct sequencing to investigate the chemical modifications of viral genomes, contributing to a deeper understanding of viral epigenetics.

Internal m^6^A was identified in herpes simplex virus (HSV) mRNA in 1977, but its relevance to viral replication was unknown [113]. By nanopore direct RNA sequencing of cells infected with HSV-1 WT or HSV-1 ΔICP27, it was observed that the expression of ICP27, a protein encoded by HSV-1, could suppress m^6^A and other RNA modifications [114]. What is more, the depletion of m^6^A methyltransferase subunits affected HSV-1 gene expression during the first 6 h of the replication cycle, indicating an important role of m^6^A in the early stages of HSV-1 infection.

The METTL3-dependent m^6^A modifications with the most frequent motif DRACH were identified within individual adenovirus transcripts at nucleotide resolution by a combination of MeRIP-seq and direct RNA long-read sequencing [24]. This study has demonstrated that m^6^A is not essential for the early stages of adenovirus infection, but the loss of m^6^A caused by the depletion of METTL3 results in the significant decrease of viral late RNAs, late proteins, and infectious progeny production. Additionally, m^6^A modifications positively regulate the splicing of viral late transcripts.

In recent years, an increasing number of studies have employed nanopore sequencing to investigate epigenetic modifications of coronavirus RNA. Consistent patterns of m^5^C methylation have been observed in different human coronavirus (HCoV)-229E transcripts through nanopore RNA sequencing, suggesting that the methylation of coronavirus RNAs is sequence-specific [22]. In 2020, researchers detected at least 41 RNA modifications on SARS-CoV-2 transcripts using nanopore direct RNA sequencing, with the most frequent motif being AAGAA [25]. Furthermore, evidence suggests that the modified viral RNAs possess shorter poly (A) tails. This finding provides new insights into the life cycle and pathogenicity of SARS-CoV-2. When combined with MeRIP-seq and nanopore direct RNA sequencing, researchers have established that β-coronavirus RNAs, including SARS-CoV-2 and human β-coronavirus HCoV-OC43, are modified with m^6^A. The replication of β-coronaviruses is controlled by host nuclear m^6^A factors, including the m^6^A methyltransferase METTL3, and two cytoplasmic m^6^A recognition proteins, YTHDF1 and YTHDF3 [115]. These findings offer a novel perspective to explore therapeutic approaches for controlling coronavirus infection.

The first comprehensive mapping of methylation cytosine in the porcine reproductive and respiratory syndrome virus (PRRSV) epitranscriptome during early infection was achieved by nanopore-based direct RNA-sequencing in 2021 [116]. The research uncovered widespread presence of m^5^C in the poly (A) RNA of two different PRRSV genotypes and significantly different quantities and distribution patterns of m^5^C. What is more, the study indicated a preferential enrichment of m^5^C modifications around the translational start codons of sgmRNAs, establishing a connection between methylation modifications and transcriptional regulation. This finding holds profound implications for the evolution and pathogenicity of PRRSV.

## 4. Concluding Remarks

Viruses, as an important class of pathogenic microorganisms, pose a serious threat to human and animal health. A number of variations, such as mutations, recombination and base deletions, often occur in the virus genome, which result in the acceleration of adaptive evolution, enhancement of immune evasion, infection of new hosts, and even the emergence of novel viruses [117,118,119]. Consequently, the timely identification of viruses, a comprehensive understanding of their genomic structures, and real-time monitoring of their genetic evolutionary characteristics are of great significance to research on viral evolution and transmission pathways, preventing disease outbreaks, and guiding clinical treatment strategies. However, conventional approaches and NGS often have limitations, such as being time-consuming, requiring high input, having complicated processes, being costly, and producing short reads, which restrict on-site detection and widespread in-depth studies of viruses.

Nanopore sequencing technology has emerging as a powerful and promising alternative in the field of virus research. It requires only a few minutes to prepare a library before sequencing and can provide sequencing data more quickly than NGS, allowing for the rapid acquisition of viral sequences. Nanopore direct sequencing is extensively used to characterize full-length transcriptomes and complex transcriptional events, identify various structure variants, and detect epigenetic marks and chemical modifications, offering numerous new insights into the pathogenic mechanisms and transmission dynamics of viruses. Due to its advantages of real-time analysis, portability, efficiency, and long-read sequencing, nanopore sequencing technology is highly favored for on-site detecting, monitoring, and analysis of viruses.

Nanopore sequencing technology represents a significant breakthrough in the field of genomics. However, despite its advancements, certain challenges persist. First, the technology exhibits an error rate that is higher compared with NGS. Current studies indicate error rates range from 5% to 20% [61,62,120,121], influenced by library preparation and the nature of molecules being sequenced [122]. For instance, the error rate of R9.4 flow cell and earlier-version flow cells that use 1D chemistry is about 10% [35]. Additionally, there is evidence to suggest that error rates are particularly elevated within G/C mononucleotide repeats [120,123]. The predominant errors include insertions and deletions at nucleotide sites [124]. In recent years, ONT has been making efforts to improve the read accuracy of nanopore sequencing. It has introduced new sequencing kits, such as Q20+, which employ a new chemistry have been successfully trialed in nanopore flow cells, reportedly boosting accuracy to 99.3% (https://nanoporetech.com/platform/accuracy/simplex (accessed on 1 January 2024)). Platforms such as the PromethION have been brought to the market, boasting sequencing accuracy rates reaching up to 98.3%. In addition, the development and application of new basecalling algorithms have significantly improved the accuracy of nanopore sequencers. Algorithms like Bonito ensure a consensus between two neural networks through alignment of their probability profiles, which effectively reduces the median sequencing error by more than half and achieves an accuracy rate of 98.3% [125]. Nevertheless, the improved accuracy of nanopore sequencing over long or ultra-long molecules is particularly commendable when compared to the error rate of short NGS sequencing, which has a maximum capacity of only 300 base pairs.

The second challenge lies in the substantial quantity of input DNA and RNA necessary for nanopore sequencing, which may amount to several micrograms of DNA and hundreds of nanograms of RNA. Particularly, for direct RNA sequencing, a large amount of starting RNA (>10 μg) is required to yield enough mRNA (500 ng) after effective rRNA depletion. In certain cases, direct amplification of viral DNA and RNA is not recommended for long-read sequencing due to the excessive PCR bias and the consequent diminution of virus representation in the sequencing data. However, for diagnostic or identification of viruses, PCR steps are optional in the library preparation for nanopore sequencing, for example sequence-independent single-primer amplification (SISPA) [49]. When detecting a viral genome, it is also advisable to conduct preliminary experiments to enrich the viral genetic material. This can be achieved through methods such as sample filtration and centrifugation, followed by treatment with DNase and RNase to remove eukaryotic and bacterial contaminants prior to nucleic acid extraction. Alternatively, ribosomal depletion targeting both eukaryotic and bacterial rRNAs can be utilized. Typically, the amount of input material often depends on the specific subject of study.

The third challenge lies in the subsequent data analysis following the acquisition of massive sequences, which are the same with the other high-throughput platforms. While numerous analysis software have been developed for nanopore data, their operation is often confined to Linux environments, which requires a degree of expertise in command-line interfaces and coding to execute analytical tasks. This presents a barrier to users without a technical background. Fortunately, ONT has launched EPI2ME (https://labs.epi2me.io (accessed on 1 January 2024)) in 2020, a comprehensive bioinformatics platform for the analysis of nanopore datasets. It offers a range of functionalities, including reference sequence alignment, metagenomic species identification, and structural variation analysis. Notably, EPI2ME is designed to be user-friendly for researchers who lack extensive computational expertise and wish to analyze nanopore data without extensive bioinformatics knowledge.

One of the common issues of nanopore sequencing is the loss of 5′ ends during direct RNA sequencing. This is probably induced by the premature release of mRNA molecules by motor proteins, which leads to a loss of control to guide the RNA through the nanopore [100]. The absence of 5′ ends hinders the accurate identification of transcriptional start sites (TSSs) and the reconstruction of the transcriptome structure. Therefore, integrating datasets from multiple sequencing platforms can aid in predicting putative 5′ ends, thereby enhancing the reliability and accuracy of the sequencing results. The nanopore community is also endeavoring to improve 5′ end detection in DRS following 5′-dependent adaptor ligation [126].

Another common issue is the template switching in certain library preparation techniques. During the transcription of RNA, template switching events caused by the reverse transcriptase (RT) can result in the production of chimeric cDNAs and artifactual introns [127]. These introns are often mistaken for splice isoforms, which adversely affects the analysis of transcripts. Direct RNA sequencing can avoid template-switching issues, thus it is crucial to select the most suitable library construction approach according to experimental needs.

Although certain challenges need to be surmounted, nanopore sequencing offers numerous benefits and holds a significant promise for advancing animal and human virus research. This technique is capable of generating extremely long reads, and providing insight into a variety of biological polymers, including DNA, RNA, and proteins. These unique features will markedly improve the continuity and integrity of genome assemblies for viruses, thus facilitating a deeper and more comprehensive understanding of the origin and evolution of viruses.

## Figures and Tables

**Table 1:** The DNA sequencing kits for nanopore sequencing.

Sequencing Kit	Advantage	Preparation Time	Input Type	Fragmentation	Parallel Quantities	Application
Ligation Sequencing Kit	Higher output	60 min	gDNA Amplified-DNA	Optional	24/96	Whole-genome sequencing/ Methylation/ Adaptive sampling
Rapid Sequencing Kit	Higher speed	10 min	gDNA	Transposase- based	24/96	Whole-genome sequencing/ Methylation/ Adaptive sampling
Rapid PCR Barcoding Kit	Lower input	15 min + PCR	gDNA	Transposase- based	24	Whole-genome sequencing/ Adaptive sampling
Ultra-Long DNA Sequencing Kit	Ultra-long reads	200 min + 1xO/N incubation	Cells	Transposase- based	-	Whole-genome sequencing/ Methylation/ Adaptive sampling
16S Barcoding Kit	Targeted sequencing	10 min + PCR	gDNA	-	24	16S sequencing/ Genus-level bacterial identification
Cas9 Sequencing Kit		110 min	gDNA	Cas9-dependent cleavage	-	Target-site sequencing/ Methylation

**Table 2:** The RNA sequencing kits for nanopore sequencing.

Type	Sequencing Kit	Preparation Time	Input Type	Fragmentation	Parallel Quantities	Application
Direct RNA	Direct RNA Sequencing Kit	105 min	500 ng total RNA 50 ng poly(A)+ RNA	Optional	-	Characterize and quantify full-length RNA transcripts, splice variants, and fusions Methylation
cDNA	cDNA-PCR Sequencing Kit	210 min + PCR	200 ng total RNA 4 ng poly(A)+ RNA		24	Characterize and quantify full-length RNA transcripts, splice variants, and fusions

**Table 3:** The software for analyzing nanopore sequencing data of viruses.

Type	Software	Functions	Availability
Sequencing	MinKNOW (Oxford Nanopore Technologies, Oxford, England)	MinKNOW is unique control software from ONT. It is used for sequencing, real-time data monitoring and simple analysis.	https://nanoporetech.com (accessed on 1 January 2024)
Basecalling	Guppy (Oxford Nanopore Technologies, Oxford, England)	Basecalling	https://nanoporetech.com (accessed on 1 January 2024)
Basecalling	Dorado (Oxford Nanopore Technologies, Oxford, England)	Dorado is a high-performance, easy-to-use, open source basecaller for Oxford Nanopore reads. It is designed for duplex basecalling, simplex barcode classification, initial support for poly(A) tail estimation, and so on.	https://github.com/nanoporetech/dorado (accessed on 1 January 2024)
Filter	Porechop version 0.2.4	Porechop is used for adapter removal and demultiplexing of nanopore reads.	https://github.com/rrwick/Porechop (accessed on 1 January 2024)
Assemble	Spades version 3.15.5	SPAdes is an assembly toolkit containing various assembly pipelines. Spades is used for bacteria, fungal, virus, and other small genomes.	https://github.com/ablab/spades (accessed on 1 January 2024)
Assemble	Canu version 2.2	Canu is an assembly toolkit designed for high-noise single-molecule sequencing. Canu includes four steps: (1) detecting overlaps in high-noise sequences, (2) generating corrected sequence consensus, (3) trimming corrected sequences, and (4) assembling trimmed corrected sequences.	https://github.com/marbl/canu (accessed on 1 January 2024)
Error correction	Medaka version 1.11.3 (Oxford Nanopore Technologies, Oxford, England)	A toolkit for polishing consensus sequences and variant calling.	https://github.com/nanoporetech/medaka (accessed on 1 January 2024)
Error correction	Pilon version 1.24	Pilon is an automated genome assembly improvement and variant detection tool.	https://github.com/broadinstitute/pilon (accessed on 1 January 2024)
Alignment	Minimap2 version 2.28	Minimap2 is a versatile sequence alignment program that aligns genomic and spliced nucleotide sequences against a large reference database.	https://github.com/lh3/minimap2 (accessed on 1 January 2024)
Alignment	Megalodon (Oxford Nanopore Technologies, Oxford, England)	Megalodon is a research command line tool for reference genome/transcriptome anchored modified base and variant analysis.	https://github.com/nanoporetech/megalodon (accessed on 1 January 2024)
Massaging	Samtools version 1.20	A tool for dealing with SAM, BAM, and CRAM files. These file formats convert to each other.	https://github.com/samtools/samtools (accessed on 1 January 2024)
Taxonomy	Centrifuge version 0.32.2	Centrifuge is a classifier for metagenomic sequence, enabling rapid, accurate, and sensitive labeling of reads and quantification of species.	https://github.com/DaehwanKimLab/centrifuge (accessed on 1 January 2024)
Taxonomy	Emu	An approach that employs an expectation-maximization (EM) algorithm to generate taxonomic abundance profiles from full-length 16S rRNA reads. This software can reduce the number of false positives and distinguish between genomically similar species.	https://github.com/treangenlab/emu (accessed on 1 January 2024)
Variant analysis	NanoVar version 1.6.2 (Oxford Nanopore Technologies, Oxford, England)	NanoVar is a genomic structural variant (SV) caller that utilizes low-depth long-read sequencing such as ONT. It characterizes six classes of SVs including novel-sequence insertion, deletion, inversion, tandem duplication, sequence transposition (TPO), and translocation (TRA), and requires 4x and 8x sequencing depth for detecting homozygous and heterozygous SVs, respectively.	https://github.com/cytham/nanovar (accessed on 1 January 2024)
Methylation analysis	Tombo version 1.5.1 (Oxford Nanopore Technologies, Oxford, England)	Tombo is a suite of tools primarily for the identification and visualization of modified nucleotides from raw nanopore sequencing data.	https://nanoporetech.github.io/tombo (accessed on 1 January 2024)
Methylation analysis	Nanopolish (Oxford Nanopore Technologies, Oxford, England)	Nanopolish is a comprehensive tool. It can calculate an improved consensus sequence for a genome assembly, detect base modifications, and call SNPs and indels with respect to a reference genome.	https://github.com/jts/nanopolish (accessed on 1 January 2024)
Methylation analysis	DRUMMER	DRUMMER is a tool to identify RNA modifications at nucleotide-level resolution on distinct transcript isoforms through the comparative analysis of basecall errors in nanopore direct RNA sequencing (DRS) datasets.	https://github.com/DepledgeLab/DRUMMER (accessed on 1 January 2024)
Sequence splitting and merging	Ont_fast5_api (Oxford Nanopore Technologies, Oxford, England)	The ont_fast5_api provides a simple interface to convert between files in the Oxford Nanopore single_read and multi_read. fast5 file formats.	https://github.com/nanoporetech/ont_fast5_api (accessed on 1 January 2024)

## Data Availability

No new data were created or analyzed in this study.

## References

[1] Zhou X., Li N., Luo Y., Liu Y., Miao F., Chen T., Zhang S., Cao P., Li X., Tian K. (2018). Emergence of African Swine Fever in China, 2018. Transbound. Emerg. Dis..

[2] Lunney J.K., Benfield D.A., Rowland R.R. (2010). Porcine reproductive and respiratory syndrome virus: An update on an emerging and re-emerging viral disease of swine. Virus Res..

[3] Huang Y.W., Dickerman A.W., Pineyro P., Li L., Fang L., Kiehne R., Opriessnig T., Meng X.J. (2013). Origin, evolution, and genotyping of emergent porcine epidemic diarrhea virus strains in the United States. mBio.

[4] Sun R.Q., Cai R.J., Chen Y.Q., Liang P.S., Chen D.K., Song C.X. (2012). Outbreak of porcine epidemic diarrhea in suckling piglets, China. Emerg. Infect. Dis..

[5] Dimitrov K.M., Ramey A.M., Qiu X., Bahl J., Afonso C.L. (2016). Temporal, geographic, and host distribution of avian paramyxovirus 1 (Newcastle disease virus). Infect. Genet. Evol..

[6] Raj G.D., Jones R.C. (1997). Infectious bronchitis virus: Immunopathogenesis of infection in the chicken. Avian Pathol..

[7] Wang N., Zhao D., Wang J., Zhang Y., Wang M., Gao Y., Li F., Wang J., Bu Z., Rao Z. (2019). Architecture of African swine fever virus and implications. Science.

[8] Zheng Y., Li S., Li S.H., Yu S., Wang Q., Zhang K., Qu L., Sun Y., Bi Y., Tang F. (2022). Transcriptome profiling in swine macrophages infected with African swine fever virus at single-cell resolution. Proc. Natl. Acad. Sci. USA.

[9] te Velthuis A.J., Fodor E. (2016). Influenza virus RNA polymerase: Insights into the mechanisms of viral RNA synthesis. Nat. Rev. Microbiol..

[10] Hutchinson E.C. (2018). Influenza Virus. Trends Microbiol..

[11] Tali S.H.S., LeBlanc J.J., Sadiq Z., Oyewunmi O.D., Camargo C., Nikpour B., Armanfard N., Sagan S.M., Jahanshahi-Anbuhi S. (2021). Tools and Techniques for Severe Acute Respiratory Syndrome. Clin. Microbiol. Rev..

[12] Younis M.C. (2021). Evaluation of deep learning approaches for identification of different corona-virus species and time series prediction. Comput. Med. Imaging Graph..

[13] Halfmann P.J., Hatta M., Chiba S., Maemura T., Fan S., Takeda M., Kinoshita N., Hattori S.-i., Sakai-Tagawa Y., Iwatsuki-Horimoto K. (2020). Transmission of SARS-CoV-2 in Domestic Cats. N. Engl. J. Med..

[14] Sit T.H.C., Brackman C.J., Ip S.M., Tam K.W.S., Law P.Y.T., To E.M.W., Yu V.Y.T., Sims L.D., Tsang D.N.C., Chu D.K.W. (2020). Canine SARS-CoV-2 infection. Nature.

[15] McAloose D., Laverack M., Wang L., Killian M.L., Caserta L.C., Yuan F., Mitchell P.K., Queen K., Mauldin M.R., Cronk B.D. (2020). From People toPanthera: Natural SARS-CoV-2 Infection in Tigers and Lions at the Bronx Zoo. mBio.

[16] Kim Y.-I., Kim S.-G., Kim S.-M., Kim E.-H., Park S.-J., Yu K.-M., Chang J.-H., Kim E.J., Lee S., Casel M.A.B. (2020). Infection and Rapid Transmission of SARS-CoV-2 in Ferrets. Cell Host Microbe.

[17] Jain M., Olsen H.E., Paten B., Akeson M. (2016). The Oxford Nanopore MinION: Delivery of nanopore sequencing to the genomics community. Genome Biol..

[18] Kilianski A., Haas J.L., Corriveau E.J., Liem A.T., Willis K.L., Kadavy D.R., Rosenzweig C.N., Minot S.S. (2015). Bacterial and viral identification and differentiation by amplicon sequencing on the MinION nanopore sequencer. GigaScience.

[19] Greninger A.L., Naccache S.N., Federman S., Yu G., Mbala P., Bres V., Stryke D., Bouquet J., Somasekar S., Linnen J.M. (2015). Rapid metagenomic identification of viral pathogens in clinical samples by real-time nanopore sequencing analysis. Genome Med..

[20] Davidson A.D., Williamson M.K., Lewis S., Shoemark D., Carroll M.W., Heesom K.J., Zambon M., Ellis J., Lewis P.A., Hiscox J.A. (2020). Characterisation of the transcriptome and proteome of SARS-CoV-2 reveals a cell passage induced in-frame deletion of the furin-like cleavage site from the spike glycoprotein. Genome Med..

[21] Tham C.Y., Tirado-Magallanes R., Goh Y., Fullwood M.J., Koh B.T.H., Wang W., Ng C.H., Chng W.J., Thiery A., Tenen D.G. (2020). NanoVar: Accurate characterization of patients’ genomic structural variants using low-depth nanopore sequencing. Genome Biol..

[22] Viehweger A., Krautwurst S., Lamkiewicz K., Madhugiri R., Ziebuhr J., Hölzer M., Marz M. (2019). Direct RNA nanopore sequencing of full-length coronavirus genomes provides novel insights into structural variants and enables modification analysis. Genome Res..

[23] Wongsurawat T., Jenjaroenpun P., Taylor M.K., Lee J., Tolardo A.L., Parvathareddy J., Kandel S., Wadley T.D., Kaewnapan B., Athipanyasilp N. (2019). Rapid Sequencing of Multiple RNA Viruses in Their Native Form. Front. Microbiol..

[24] Price A.M., Hayer K.E., McIntyre A.B.R., Gokhale N.S., Abebe J.S., Della Fera A.N., Mason C.E., Horner S.M., Wilson A.C., Depledge D.P. (2020). Direct RNA sequencing reveals m^6^A modifications on adenovirus RNA are necessary for efficient splicing. Nat. Commun..

[25] Kim D., Lee J.-Y., Yang J.-S., Kim J.W., Kim V.N., Chang H. (2020). The Architecture of SARS-CoV-2 Transcriptome. Cell.

[26] Faria N.R., Quick J., Claro I.M., Thézé J., de Jesus J.G., Giovanetti M., Kraemer M.U.G., Hill S.C., Black A., da Costa A.C. (2017). Establishment and cryptic transmission of Zika virus in Brazil and the Americas. Nature.

[27] Lu R., Zhao X., Li J., Niu P., Yang B., Wu H., Wang W., Song H., Huang B., Zhu N. (2020). Genomic characterisation and epidemiology of 2019 novel coronavirus: Implications for virus origins and receptor binding. Lancet.

[28] Boykin L.M., Sseruwagi P., Alicai T., Ateka E., Mohammed I.U., Stanton J.-A.L., Kayuki C., Mark D., Fute T., Erasto J. (2019). Tree Lab: Portable genomics for Early Detection of Plant Viruses and Pests in Sub-Saharan Africa. Genes.

[29] Roach N.P., Sadowski N., Alessi A.F., Timp W., Taylor J., Kim J.K. (2020). The full-length transcriptome of C. elegans using direct RNA sequencing. Genome Res..

[30] Euskirchen P., Bielle F., Labreche K., Kloosterman W.P., Rosenberg S., Daniau M., Schmitt C., Masliah-Planchon J., Bourdeaut F., Dehais C. (2017). Same-day genomic and epigenomic diagnosis of brain tumors using real-time nanopore sequencing. Acta Neuropathol..

[31] De Roeck A., De Coster W., Bossaerts L., Cacace R., De Pooter T., Van Dongen J., D’Hert S., De Rijk P., Strazisar M., Van Broeckhoven C. (2019). NanoSatellite: Accurate characterization of expanded tandem repeat length and sequence through whole genome long-read sequencing on PromethION. Genome Biol..

[32] Wang Y., Zhao Y., Bollas A., Wang Y., Au K.F. (2021). Nanopore sequencing technology, bioinformatics and applications. Nat. Biotechnol..

[33] Petersen L.M., Martin I.W., Moschetti W.E., Kershaw C.M., Tsongalis G.J. (2019). Third-Generation Sequencing in the Clinical Laboratory: Exploring the Advantages and Challenges of Nanopore Sequencing. J. Clin. Microbiol..

[34] Quick J., Loman N.J., Duraffour S., Simpson J.T., Severi E., Cowley L., Bore J.A., Koundouno R., Dudas G., Mikhail A. (2016). Real-time, portable genome sequencing for Ebola surveillance. Nature.

[35] Lu H., Giordano F., Ning Z. (2016). Oxford Nanopore MinION Sequencing and Genome Assembly. Genom. Proteom. Bioinform..

[36] Deamer D., Akeson M., Branton D. (2016). Three decades of nanopore sequencing. Nat. Biotechnol..

[37] Kasianowicz J.J., Brandin E., Branton D., Deamer D.W. (1996). Characterization of individual polynucleotide molecules using a membrane channel. Proc. Natl. Acad. Sci. USA.

[38] Akeson M., Branton D., Kasianowicz J.J., Brandin E., Deamer D.W. (1999). Microsecond Time-Scale Discrimination Among Polycytidylic Acid, Polyadenylic Acid, and Polyuridylic Acid as Homopolymers or as Segments Within Single RNA Molecules. Biophys. J..

[39] Ashkenasy N., Sánchez-Quesada J., Bayley H., Ghadiri M.R. (2005). Recognizing a single base in an individual DNA strand: A step toward DNA sequencing in nanopores. Angew. Chem. Int. Ed. Engl..

[40] Stoddart D., Heron A.J., Mikhailova E., Maglia G., Bayley H. (2009). Single-nucleotide discrimination in immobilized DNA oligonucleotides with a biological nanopore. Proc. Natl. Acad. Sci. USA.

[41] Stoddart D., Heron A.J., Klingelhoefer J., Mikhailova E., Maglia G., Bayley H. (2010). Nucleobase Recognition in ssDNA at the Central Constriction of the α-Hemolysin Pore. Nano Lett..

[42] Stoddart D., Maglia G., Mikhailova E., Heron A.J., Bayley H. (2010). Multiple Base-Recognition Sites in a Biological Nanopore: Two Heads are Better than One. Angew. Chem. Int. Ed..

[43] Manrao E.A., Derrington I.M., Laszlo A.H., Langford K.W., Hopper M.K., Gillgren N., Pavlenok M., Niederweis M., Gundlach J.H. (2012). Reading DNA at single-nucleotide resolution with a mutant MspA nanopore and phi29 DNA polymerase. Nat. Biotechnol..

[44] Leggett R.M., Clark M.D. (2017). A world of opportunities with nanopore sequencing. J. Exp. Bot..

[45] Jain M., Fiddes I.T., Miga K.H., E Olsen H., Paten B., Akeson M. (2015). Improved data analysis for the MinION nanopore sequencer. Nat. Methods.

[46] Zwolak M., Di Ventra M. (2005). Electronic Signature of DNA Nucleotides via Transverse Transport. Nano Lett..

[47] Lin B., Hui J., Mao H. (2021). Nanopore Technology and Its Applications in Gene Sequencing. Biosensors.

[48] Mohammad M.M., Iyer R., Howard K.R., McPike M.P., Borer P.N., Movileanu L. (2012). Engineering a Rigid Protein Tunnel for Biomolecular Detection. J. Am. Chem. Soc..

[49] Lewandowski K., Xu Y., Pullan S.T., Lumley S.F., Foster D., Sanderson N., Vaughan A., Morgan M., Bright N., Kavanagh J. (2019). Metagenomic Nanopore Sequencing of Influenza Virus Direct. J. Clin. Microbiol..

[50] Brown E., Freimanis G., Shaw A.E., Horton D.L., Gubbins S., King D. (2021). Characterising Foot-and-Mouth Disease Virus in Clinical Samples Using Nanopore Sequencing. Front. Vet. Sci..

[51] Hu Z.L., Huo M.Z., Ying Y.L., Long Y.T. (2021). Biological Nanopore Approach for Single-Molecule Protein Sequencing. Angew. Chem. Int. Ed..

[52] Zheng P., Zhou C., Ding Y., Liu B., Lu L., Zhu F., Duan S. (2023). Nanopore sequencing technology and its applications. MedComm.

[53] Kilianski A., Roth P.A., Liem A.T., Hill J.M., Willis K.L., Rossmaier R.D., Marinich A.V., Maughan M.N., Karavis M.A., Kuhn J.H. (2016). Use of Unamplified RNA/cDNA-Hybrid Nanopore Sequencing for Rapid Detection and Characterization of RNA Viruses. Emerg. Infect. Dis..

[54] Brinkmann A., Ulm S.-L., Uddin S., Förster S., Seifert D., Oehme R., Corty M., Schaade L., Michel J., Nitsche A. (2021). AmpliCoV: Rapid Whole-Genome Sequencing Using Multiplex PCR Amplification and Real-Time Oxford Nanopore MinION Sequencing Enables Rapid Variant Identification of SARS-CoV-2. Front. Microbiol..

[55] Lee R.S., Pai M. (2017). Real-Time Sequencing of Mycobacterium tuberculosis: Are We There Yet?. J. Clin. Microbiol..

[56] Faria N.R., Sabino E.C., Nunes M.R.T., Alcantara L.C.J., Loman N.J., Pybus O.G. (2016). Mobile real-time surveillance of Zika virus in Brazil. Genome Med..

[57] Castro-Wallace S.L., Chiu C.Y., John K.K., Stahl S.E., Rubins K.H., McIntyre A.B.R., Dworkin J.P., Lupisella M.L., Smith D.J., Botkin D.J. (2017). Nanopore DNA Sequencing and Genome Assembly on the International Space Station. Sci. Rep..

[58] McIntyre A.B.R., Rizzardi L., Yu A.M., Alexander N., Rosen G.L., Botkin D.J., E Stahl S., John K.K., Castro-Wallace S.L., McGrath K. (2016). Nanopore sequencing in microgravity. NPJ Microgravity.

[59] Payne A., Holmes N., Rakyan V., Loose M., Birol I. (2019). BulkVis: A graphical viewer for Oxford nanopore bulk FAST5 files. Bioinformatics.

[60] Gong L., Wong C.-H., Cheng W.-C., Tjong H., Menghi F., Ngan C.Y., Liu E.T., Wei C.-L. (2018). Picky comprehensively detects high-resolution structural variants in nanopore long reads. Nat. Methods.

[61] Stancu M.C., van Roosmalen M.J., Renkens I., Nieboer M.M., Middelkamp S., de Ligt J., Pregno G., Giachino D., Mandrile G., Valle-Inclan J.E. (2017). Mapping and phasing of structural variation in patient genomes using nanopore sequencing. Nat. Commun..

[62] Jain M., Koren S., Miga K.H., Quick J., Rand A.C., Sasani T.A., Tyson J.R., Beggs A.D., Dilthey A.T., Fiddes I.T. (2018). Nanopore sequencing and assembly of a human genome with ultra-long reads. Nat. Biotechnol..

[63] Nurk S., Koren S., Rhie A., Rautiainen M., Bzikadze A.V., Mikheenko A., Vollger M.R., Altemose N., Uralsky L., Gershman A. (2022). The complete sequence of a human genome. Science.

[64] Loose M., Malla S., Stout M. (2016). Real-time selective sequencing using nanopore technology. Nat. Methods.

[65] Ranasinghe D., Jayadas T.T.P., Jayathilaka D., Jeewandara C., Dissanayake O., Guruge D., Ariyaratne D., Gunasinghe D., Gomes L., Wijesinghe A. (2022). Comparison of different sequencing techniques for identification of SARS-CoV-2 variants of concern with multiplex real-time PCR. PLoS ONE.

[66] Logsdon G.A., Vollger M.R., Eichler E.E. (2020). Long-read human genome sequencing and its applications. Nat. Rev. Genet..

[67] Parker J., Helmstetter A.J., Devey D., Wilkinson T., Papadopulos A.S.T. (2017). Field-based species identification of closely-related plants using real-time nanopore sequencing. Sci. Rep..

[68] Edwards A., Debbonaire A.R., Nicholls S.M., Rassner S.M.E., Sattler B., Cook J.M., Davy T., Soares A., Mur L.A.J., Hodson A.J. (2016). In-field metagenome and 16S rRNA gene amplicon nanopore sequencing robustly characterize glacier microbiota. bioRxiv.

[69] Pomerantz A., Penafiel N., Arteaga A., Bustamante L., Pichardo F., Coloma L.A., Barrio-Amoros C.L., Salazar-Valenzuela D., Prost S. (2018). Real-time DNA barcoding in a rainforest using nanopore sequencing: Opportunities for rapid biodiversity assessments and local capacity building. GigaScience.

[70] Pomerantz A., Sahlin K., Vasiljevic N., Seah A., Lim M., Humble E., Kennedy S., Krehenwinkel H., Winter S., Ogden R. (2022). Rapid in situ identification of biological specimens via DNA amplicon sequencing using miniaturized laboratory equipment. Nat. Protoc..

[71] Egeter B., Veríssimo J., Lopes-Lima M., Chaves C., Pinto J., Riccardi N., Beja P., Fonseca N. (2022). Speeding up the detection of invasive bivalve species using environmental DNA: A Nanopore and Illumina sequencing comparison. Mol. Ecol. Resour..

[72] Wiener D., Schwartz S. (2021). The epitranscriptome beyond m^6^A. Nat. Rev. Genet..

[73] Leger A., Amaral P.P., Pandolfini L., Capitanchik C., Capraro F., Miano V., Migliori V., Toolan-Kerr P., Sideri T., Enright A.J. (2021). RNA modifications detection by comparative Nanopore direct RNA sequencing. Nat. Commun..

[74] Zhang X., Hao H., Ma L., Zhang Y., Hu X., Chen Z., Liu D., Yuan J., Hu Z., Guan W. (2021). Methyltransferase-like 3 Modulates Severe Acute Respiratory Syndrome Coronavirus-2 RNA N6-Methyladenosine Modification and Replication. mBio.

[75] Wang D., Jiang A., Feng J., Li G., Guo D., Sajid M., Wu K., Zhang Q., Ponty Y., Will S. (2021). The SARS-CoV-2 subgenome landscape and its novel regulatory features. Mol. Cell.

[76] Arias M., De La Torre A., Dixon L., Gallardo C., Jori F., Laddomada A., Martins C., Parkhouse R.M., Revilla Y., Rodriguez F.a.J.-M. (2017). Approaches and Perspectives for Development of African Swine Fever Virus Vaccines. Vaccines.

[77] Jia L., Jiang M., Wu K., Hu J., Wang Y., Quan W., Hao M., Liu H., Wei H., Fan W. (2020). Nanopore sequencing of African swine fever virus. Sci. China Life Sci..

[78] Forth J.H., Forth L.F., King J., Groza O., Hübner A., Olesen A.S., Höper D., Dixon L.K., Netherton C.L., Rasmussen T.B. (2019). A Deep-Sequencing Workflow for the Fast and Efficient Generation of High-Quality African Swine Fever Virus Whole-Genome Sequences. Viruses.

[79] O’Donnell V.K., Grau F.R., Mayr G.A., Samayoa T.L.S., Dodd K.A., Barrette R.W. (2019). Rapid Sequence-Based Characterization of African Swine Fever Virus by Use of the Oxford Nanopore MinION Sequence Sensing Device and a Companion Analysis Software Tool. J. Clin. Microbiol..

[80] Isidro J., Borges V., Pinto M., Sobral D., Santos J.D., Nunes A., Mixão V., Ferreira R., Santos D., Duarte S. (2022). Phylogenomic characterization and signs of microevolution in the 2022 multi-country outbreak of monkeypox virus. Nat. Med..

[81] Luna N., Muñoz M., Bonilla-Aldana D.K., Patiño L.H., Kasminskaya Y., Paniz-Mondolfi A., Ramírez J.D. (2023). Monkeypox virus (MPXV) genomics: A mutational and phylogenomic analyses of B.1 lineages. Travel Med. Infect. Dis..

[82] Luna N., Ramírez A.L., Muñoz M., Ballesteros N., Patiño L.H., Castañeda S.A., Bonilla-Aldana D.K., Paniz-Mondolfi A., Ramírez J.D. (2022). Phylogenomic analysis of the monkeypox virus (MPXV) 2022 outbreak: Emergence of a novel viral lineage?. Travel Med. Infect. Dis..

[83] Kumar N., Acharya A., Gendelman H.E., Byrareddy S.N. (2022). The 2022 outbreak and the pathobiology of the monkeypox virus. J. Autoimmun..

[84] Kakuk B., Dormo A., Csabai Z., Kemenesi G., Holoubek J., Ruzek D., Prazsak I., Dani V.E., Denes B., Torma G. (2023). In-depth Temporal Transcriptome Profiling of Monkeypox and Host Cells using Nanopore Sequencing. Sci. Data.

[85] Bosmeny M.S., White A.A., Pater A.A., Crew J., Geltz J., Gagnon K.T. (2023). Global mpox lineage discovery and rapid outbreak tracking with nanopore sequencing. Virol. J..

[86] Webby R.J., Webster R.G. (2023). Are we ready for pandemic influenza?. Science.

[87] Xu Y., Lewandowski K., Downs L.O., Kavanagh J., Hender T., Lumley S., Jeffery K., Foster D., Sanderson N.D., Vaughan A. (2021). Nanopore metagenomic sequencing of influenza virus directly from respiratory samples: Diagnosis, drug resistance and nosocomial transmission, United Kingdom, 2018/19 influenza season. Eurosurveillance.

[88] Rambo-Martin B.L., Keller M.W., Wilson M.M., Nolting J.M., Anderson T.K., Vincent A.L., Bagal U.R., Jang Y., Neuhaus E.B., Davis C.T. (2020). Influenza A Virus Field Surveillance at a Swine-Human Interface. mSphere.

[89] Williams T.G.S., Snell L.B., Alder C., Charalampous T., Alcolea-Medina A., Sehmi J.K., Al-Yaakoubi N., Humayun G., Miah S., Lackenby A. (2023). Feasibility and clinical utility of local rapid Nanopore influenza A virus whole genome sequencing for integrated outbreak management, genotypic resistance detection and timely surveillance. Microb. Genom..

[90] Yip C.C.-Y., Chan W.-M., Ip J.D., Seng C.W.-M., Leung K.-H., Poon R.W.-S., Ng A.C.-K., Wu W.-L., Zhao H., Chan K.-H. (2020). Nanopore Sequencing Reveals Novel Targets for Detection and Surveillance of Human and Avian Influenza A Viruses. J. Clin. Microbiol..

[91] Xu Y., Lewandowski K., Jeffery K., Downs L.O., Foster D., Sanderson N.D., Kavanagh J., Vaughan A., Salvagno C., Vipond R. (2020). Nanopore metagenomic sequencing to investigate nosocomial transmission of human metapneumovirus from a unique genetic group among haematology patients in the United Kingdom. J. Infect..

[92] Groen K., van Nieuwkoop S., Meijer A., van der Veer B., van Kampen J.J.A., Fraaij P.L., Fouchier R.A.M., van den Hoogen B.G. (2022). Emergence and Potential Extinction of Genetic Lineages of Human Metapneumovirus between 2005 and 2021. mBio.

[93] Li J., Wang H., Mao L., Yu H., Yu X., Sun Z., Qian X., Cheng S., Chen S., Chen J. (2020). Rapid genomic characterization of SARS-CoV-2 viruses from clinical specimens using nanopore sequencing. Sci. Rep..

[94] Snell L.B., Alcolea-Medina A., Charalampous T., Alder C., Williams T.G.S., Flaviani F., Batra R., Bakrania P., Thangarajah R., Neil S.J.D. (2023). Real-Time Whole Genome Sequencing to Guide Patient-Tailored Therapy of Severe Acute Respiratory Syndrome Coronavirus 2 Infection. Clin. Infect. Dis..

[95] Løvestad A.H., Jørgensen S.B., Handal N., Ambur O.H., Aamot H.V. (2021). Investigation of intra-hospital SARS-CoV-2 transmission using nanopore whole-genome sequencing. J. Hosp. Infect..

[96] Charre C., Regue H., Dény P., Josset L., Chemin I., Zoulim F., Scholtes C. (2023). Improved hepatitis delta virus genome characterization by single molecule full-length genome sequencing combined with VIRiONT pipeline. J. Med. Virol..

[97] Zerboni L., Sen N., Oliver S.L., Arvin A.M. (2014). Molecular mechanisms of varicella zoster virus pathogenesis. Nat. Rev. Microbiol..

[98] Prazsák I., Moldován N., Balázs Z., Tombácz D., Megyeri K., Szűcs A., Csabai Z., Boldogkői Z. (2018). Long-read sequencing uncovers a complex transcriptome topology in varicella zoster virus. BMC Genom..

[99] Zheng H.H., Fu P.F., Chen H.Y., Wang Z.Y. (2022). Pseudorabies Virus: From Pathogenesis to Prevention Strategies. Viruses.

[100] Moldován N., Tombácz D., Szűcs A., Csabai Z., Snyder M., Boldogkői Z. (2017). Multi-Platform Sequencing Approach Reveals a Novel Transcriptome Profile in Pseudorabies Virus. Front. Microbiol..

[101] Rocholl C., Gerber K., Daly J., Pavia A.T., Byington C.L. (2004). Adenoviral infections in children: The impact of rapid diagnosis. Pediatrics.

[102] Lynch J., Fishbein M., Echavarria M. (2011). Adenovirus. Semin. Respir. Crit. Care Med..

[103] Berciaud S., Rayne F., Kassab S., Jubert C., Faure-Della Corte M., Salin F., Wodrich H., Lafon M.E. (2012). Adenovirus infections in Bordeaux University Hospital 2008–2010: Clinical and virological features. J. Clin. Virol..

[104] Savón C., Acosta B., Valdés O., Goyenechea A., Gonzalez G., Piñón A., Más P., Rosario D., Capó V., Kourí V. (2008). A myocarditis outbreak with fatal cases associated with adenovirus subgenera C among children from Havana City in 2005. J. Clin. Virol..

[105] Westergren Jakobsson A., Segerman B., Wallerman O., Bergström Lind S., Zhao H., Rubin C.-J., Pettersson U., Akusjärvi G., Parrish C.R. (2021). The Human Adenovirus 2 Transcriptome: An Amazing Complexity of Alternatively Spliced mRNAs. J. Virol..

[106] Price A.M., Steinbock R.T., Lauman R., Charman M., Hayer K.E., Kumar N., Halko E., Lum K.K., Wei M., Wilson A.C. (2022). Novel viral splicing events and open reading frames revealed by long-read direct RNA sequencing of adenovirus transcripts. PLoS Pathog..

[107] Ugolini C., Mulroney L., Leger A., Castelli M., Criscuolo E., Williamson M.K., Davidson A.D., Almuqrin A., Giambruno R., Jain M. (2022). Nanopore ReCappable sequencing maps SARS-CoV-2 5′ capping sites and provides new insights into the structure of sgRNAs. Nucleic Acids Res..

[108] Bull R.A., Adikari T.N., Ferguson J.M., Hammond J.M., Stevanovski I., Beukers A.G., Naing Z., Yeang M., Verich A., Gamaarachchi H. (2020). Analytical validity of nanopore sequencing for rapid SARS-CoV-2 genome analysis. Nat. Commun..

[109] Gokhale N.S., Horner S.M. (2017). RNA modifications go viral. PLoS Pathog..

[110] Courtney D.G. (2021). Post-Transcriptional Regulation of Viral RNA through Epitranscriptional Modification. Cells.

[111] Simpson J.T., Workman R.E., Zuzarte P.C., David M., Dursi L.J., Timp W. (2017). Detecting DNA cytosine methylation using nanopore sequencing. Nat. Methods.

[112] Ni P., Huang N., Zhang Z., Wang D.P., Liang F., Miao Y., Xiao C.L., Luo F., Wang J. (2019). DeepSignal: Detecting DNA methylation state from Nanopore sequencing reads using deep-learning. Bioinformatics.

[113] Moss B., Gershowitz A., Stringer J.R., E Holland L., Wagner E.K. (1977). 5′-Terminal and internal methylated nucleosides in herpes simplex virus type 1 mRNA. J. Virol..

[114] Srinivas K.P., Depledge D.P., Abebe J.S., Rice S.A., Mohr I., Wilson A.C. (2021). Widespread remodeling of the m^6^A RNA-modification landscape by a viral regulator of RNA processing and export. Proc. Natl. Acad. Sci. USA.

[115] Burgess H.M., Depledge D.P., Thompson L., Srinivas K.P., Grande R.C., Vink E.I., Abebe J.S., Blackaby W.P., Hendrick A., Albertella M.R. (2021). Targeting the m^6^A RNA modification pathway blocks SARS-CoV-2 and HCoV-OC43 replication. Genes Dev..

[116] Zhang R., Wang P., Ma X., Wu Y., Luo C., Qiu L., Zeshan B., Yang Z., Zhou Y., Wang X. (2021). Nanopore-Based Direct RNA-Sequencing Reveals a High-Resolution Transcriptional Landscape of Porcine Reproductive and Respiratory Syndrome Virus. Viruses.

[117] Malim M.H., Emerman M. (2001). HIV-1 sequence variation: Drift, shift, and attenuation. Cell.

[118] Khatchikian D., Orlich M., Rott R. (1989). Increased viral pathogenicity after insertion of a 28S ribosomal RNA sequence into the haemagglutinin gene of an influenza virus. Nature.

[119] Hon C.-C., Lam T.-Y., Shi Z.-L., Drummond A.J., Yip C.-W., Zeng F., Lam P.-Y., Leung F.C.-C. (2008). Evidence of the Recombinant Origin of a Bat Severe Acute Respiratory Syndrome (SARS)-Like Coronavirus and Its Implications on the Direct Ancestor of SARS Coronavirus. J. Virol..

[120] Ashton P.M., Nair S., Dallman T., Rubino S., Rabsch W., Mwaigwisya S., Wain J., O’Grady J. (2014). MinION nanopore sequencing identifies the position and structure of a bacterial antibiotic resistance island. Nat. Biotechnol..

[121] Laver T., Harrison J., O’Neill P.A., Moore K., Farbos A., Paszkiewicz K., Studholme D.J. (2015). Assessing the performance of the Oxford Nanopore Technologies MinION. Biomol. Detect. Quantif..

[122] Xu Y., Lewandowski K., Lumley S., Pullan S., Vipond R., Carroll M., Foster D., Matthews P.C., Peto T., Crook D. (2018). Detection of Viral Pathogens With Multiplex Nanopore MinION Sequencing: Be Careful With Cross-Talk. Front. Microbiol..

[123] Karlsson E., Lärkeryd A., Sjödin A., Forsman M., Stenberg P. (2015). Scaffolding of a bacterial genome using MinION nanopore sequencing. Sci. Rep..

[124] Rang F.J., Kloosterman W.P., de Ridder J. (2018). From squiggle to basepair: Computational approaches for improving nanopore sequencing read accuracy. Genome Biol..

[125] Silvestre-Ryan J., Holmes I. (2021). Pair consensus decoding improves accuracy of neural network basecallers for nanopore sequencing. Genome Biol..

[126] Grünberger F., Ferreira-Cerca S., Grohmann D. (2022). Nanopore sequencing of RNA and cDNA molecules in Escherichia coli. RNA.

[127] Cocquet J., Chong A., Zhang G., Veitia R.A. (2006). Reverse transcriptase template switching and false alternative transcripts. Genomics.

